# EHMTI-0379. Update of the uk post market pilot programme with single pulse transcranial magnetic stimulation (STMS) for the acute treatment of migraine

**DOI:** 10.1186/1129-2377-15-S1-M2

**Published:** 2014-09-18

**Authors:** R Bhola, E Kinsella, F Ahmed, PJ Goadsby

**Affiliations:** 1 Clinical Research, eNeura Therapeutics, London, UK; 2 Neurology, Hull and East Yorkshire NHS Hospitals Trust, Hull, UK; 3 Headache Group - Clinical Neuroscience, NIHR Welcome Trust Clinical Research Facility Kings College London, London, UK

## Introduction

Some patients suffer disabling, frequent migraine without effective treatment as current pharmacological options may be contra-indicated, poorly tolerated or overused. Single pulse transcranial magnetic stimulation (sTMS) is a novel, CE marked, non-drug treatment for migraine.

## Aim

To evaluate the patient response to sTMS in open outpatient settings at UK headache clinics, and to assess the impact of sTMS over three months.

## Methods

Clinicians selected patients and prescribed the device. Migraine patients with and without aura treating with sTMS had an initial review and training call (n = 304) with a headache nurse and then participated in telephone surveys at week six (n = 157) and week twelve during a 3-month treatment period (n= 122; episodic, n = 42; chronic, n = 80). Patient outcomes were documented, anonymised and analyzed and are presented here.

## Results

In total 122 (35%) patients have been using the device for a minimum of three months and completed surveys. Of these, 89 (73%) reported a reduction or alleviation of pain. 101 (83%) were also using an acute medication at the time of prescription. Of these, 69 (68%) reported a reduction in the number of days of medications use. The treatment was well tolerated with no serious or unanticipated adverse events reported.

## Conclusions

sTMS may be a valuable addition to options for the treatment of both episodic and chronic migraine. This device is safe to use in clinical practice and has reliable, reproducible effects on migraine over time.

The UK Pilot Programme was supported by eNeura Therapeutics.

